# The structure of *Chlamydomonas* LOV1 as revealed by time-resolved serial synchrotron crystallography

**DOI:** 10.1107/S2052252524008327

**Published:** 2024-08-28

**Authors:** Marius Schmidt

**Affiliations:** a Kenwood Interdisciplinary Research Complex, University of Wisconsin-Milwaukee, 3135 North Maryland Avenue, Milwaukee, WI53211, USA

**Keywords:** time-resolved serial synchrotron crystallography, TR-SSX, room-temperature crystallography, blue-light photoreceptors, *Chlamydomonas reinhardtii*, *Cr*PhotLOV1, structural dynamics, light–oxygen–voltage domains

## Abstract

The photo-reaction of the LOV1 domain of the *Chlamydomonas reinhardtii* phototropin is investigated by room-temperature time-resolved serial crystallography. A covalent adduct forms between the C4a atom of the central flavin-mononucleotide chromophore and a protein cysteine. The structure of the adduct is very similar to that of LOV2 determined 23 years ago from the maidenhair fern Phy3.

A collaboration led by Joerg Standfuss, Paul Scherer Institute in Switzerland, and Przemyslaw Nogly from the Jagiellonian University in Krakov, Poland, investigated the reaction of a blue light photoreceptor domain derived from the phototropin of a single cellular organism. Results are published in an article in this issue of **IUCrJ** (Gotthard *et al.*, 2024 ▸). Phototropins are proteins found in plants, algae and fungi with homologs in bacteria. They are involved in various physiological responses from growth-towards-light (phototropism) to chloro­plast light avoidance. The understanding of the light-activation reaction, and particularly that of the subsequent deactivation, plays an important role in controlling these physiological responses. Twenty-three years ago, the photoactive domain LOV2 from the maidenhair fern (*Adiantum capillus-veneris*) phytochrome/phototropin chimera Phy3 was characterized by Sean Crosson and Keith Moffat at the University of Chicago, USA (Crosson & Moffat, 2001 ▸). LOV (light–oxygen–voltage) domains are found in receptor proteins through all kingdoms of life. They are typically involved in regulating enzyme activity upon receiving an outside stimulus. Some proteins containing LOV domains are also found in humans, for example in cardiac potassium channels.

Phototropin LOV domains (LOV1 and LOV2) contain a flavin-mononucleotide (FMN) chromophore, each, that absorbs light in the blue–green region. Importantly, when light is absorbed, one of the FMN carbons (C4a) forms a covalent bond with a nearby cysteine residue of the LOV protein. For the fern LOV2 two different structures were determined, one in the dark and one under continuous light illumination (Crosson & Moffat, 2001 ▸, 2002 ▸). A few years later, Fedorov *et al.* extended these investigations to LOV1 of the phototropin from the single-cell green algae *Chlamydomonas reinhardtii* (Fedorov *et al.*, 2003 ▸). *Chlamydomonas* is biologically highly significant as a basic model for a photosynthetic organism.

In the study by Gotthard *et al.*, the photoreaction of the *Chlamydomonas* LOV1 was re-examined. The experiments were performed with time-resolved serial synchrotron crystallography (TR-SSX), where a large number of small crystals are transported into the X-ray interaction volume by a (viscous) jet and exposed one by one to intense X-ray radiation. However, before the crystals are probed by X-rays they are excited by a 5 ms blue–green (λ = 488 nm) light pulse from a laser diode. The data were collected in a stroboscopic way. This means that once a jet volume containing crystals was excited, multiple exposures to X-ray pulses and corresponding detector readouts were recorded at increasing time delays. The serial crystallography approach has the advantage that radiation damage is minimized (although not avoided), which has been a potential issue with previous investigations on LOV proteins.

Since the yield of the excited photoproduct is low, multiple absorption events are particularly important to repeatedly activate the LOV1 to boost population transfer. The authors report six activation events on average during the 5 ms laser-light exposure. This results in difference electron density maps of exquisite quality, which could be easily interpreted. Fig. 1 ▸ shows the structure of LOV1 2.5 ms after light activation. The structure of the cysteinyl-FMN thio­ether covalent adduct is clearly visible. Upon adduct formation, the C4a carbon configuration changes from a planar *sp*^2^ to a more tetrahedral *sp*^3^-hybridized configuration, thereby distorting the structure of the isoalloxazine moiety of the FMN. The orientation of Cys52 is decisively different from that determined originally by Fedorov *et al.* It agrees more with the orientation observed by Crosson and Moffat earlier.

The paper also reports an important lesson learned when approximately Gaussian laser-light intensity profiles are employed together with stroboscopic data collection. For practical reasons, the fastest time point, which was the 2.5 ms time point in this case, should be excited by the maximum of the intensity profile. Crystals probed at later time points are transported a longer distance by the viscous jet into the X-ray interaction volume. These crystals are excited by subsequently smaller intensities from the flanks of the light intensity profile. This coercively means that the extent of population transfer to the covalently bound thio­ether adduct is smaller at later time points. The covalently bound adduct has a lifetime of several 100 s, yet the timescale probed in the paper is 400 ms (80 X-ray exposures for 5 ms each). On the measured timescale, a decay of the adduct species cannot be determined, yet an apparent decay of more than 70% is reported already at a pump–probe delay of 90 ms. This behavior can only be attributed to a smaller effective population transfer caused by less laser-light intensity from the flanks of the intensity profile. In addition, the 2.5 ms time point does not show the largest activation level. In fact, the largest concentration of the covalent adduct is observed at 22 ms. This means that the 22 ms time point was activated by the peak of the light intensity distribution and the 2.5 ms time point was excited by a slightly less, off-peak intensity. Nevertheless, important structural relaxations were discovered at time points longer than 32 ms after adduct formation as demonstrated by 19 time-resolved structures (up to 93 ms) deposited in the Protein Data Bank.

In summary, Gotthard *et al.* investigated the PHY1 activated state at numerous time delays, but with varying activation levels caused by different pump laser-light intensities. The structure of the FMN thio­ether covalent adduct determined by TR-SSX is different from that determined at cryo-temperatures. The results highlight the significance of room-temperature investigations in conjunction with SSX as an effective method to reduce radiation damage during data collection by distributing the X-ray dose over a large number of protein crystals.

## Figures and Tables

**Figure 1:**
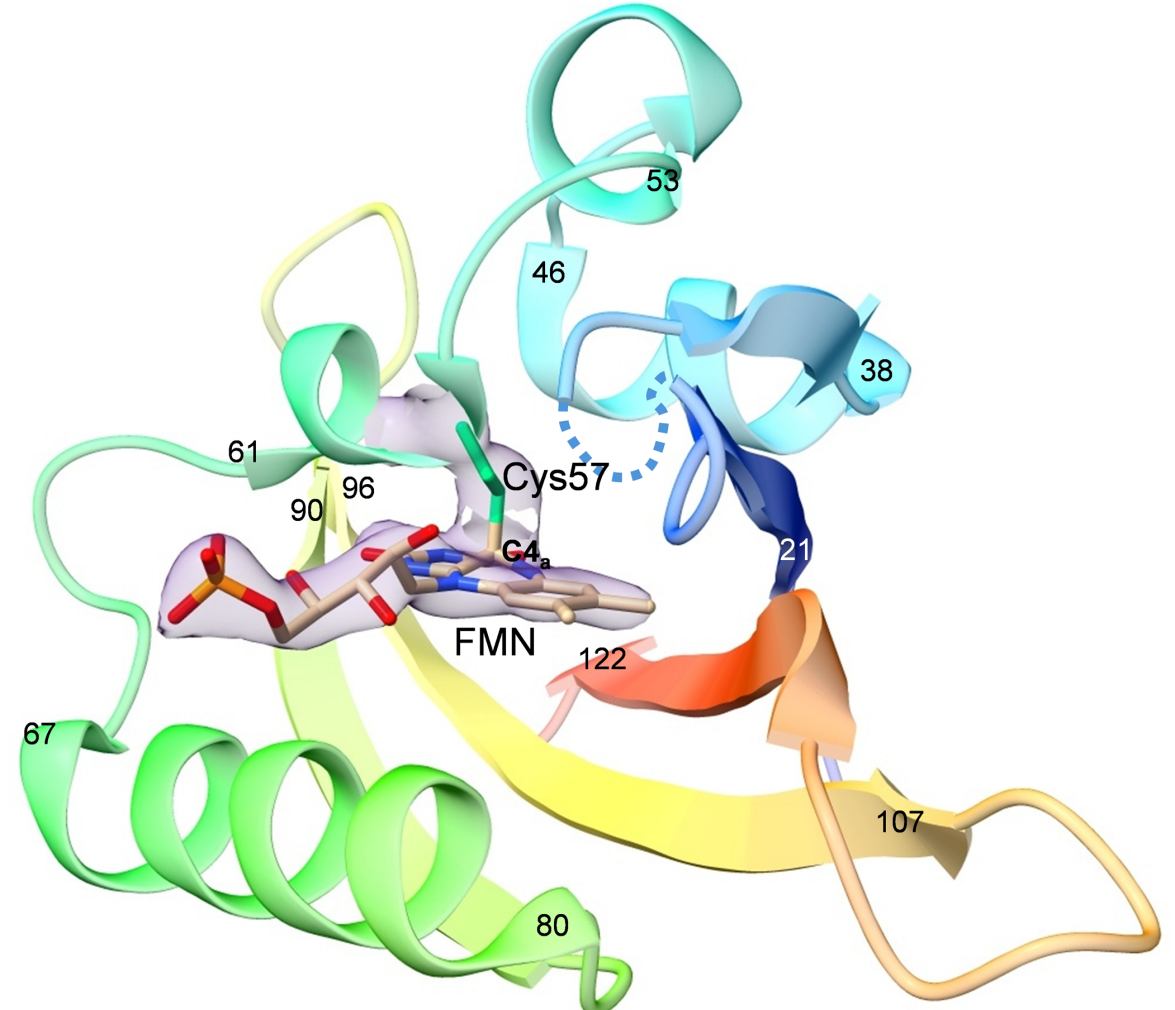
Structure of *Chlamydomonas* LOV1 2.5 ms after light activation. The FMN-Cys57 thio­ether covalent adduct is clearly visible in the electron density (transparent blue). The FMN and Cys57 are marked. Residue numbers are marked (from 21 to 122) to help tracing the amino acid backbone from the N- to the C-terminus. Dotted blue line: the loop is removed to allow for an unperturbed view on the FMN binding pocket. Structure and structure factors obtained from PDB entry 8qia.
